# Research on Multi-label Text Classification Method Based on tALBERT-CNN

**DOI:** 10.1007/s44196-021-00055-4

**Published:** 2021-12-13

**Authors:** Wenfu Liu, Jianmin Pang, Nan Li, Xin Zhou, Feng Yue

**Affiliations:** 1 State Key Laboratory of Mathematical Engineering and Advanced Computing, Zhengzhou, Henan China; 2 State Key Laboratory of Complex Electromagnetic Environment Effects on Electronics and Information System, Luoyang, Henan China

**Keywords:** Multi-label, Text classification, ALBERT, Topic model, Fusion mechanism

## Abstract

Single-label classification technology has difficulty meeting the needs of text classification, and multi-label text classification has become an important research issue in natural language processing (NLP). Extracting semantic features from different levels and granularities of text is a basic and key task in multi-label text classification research. A topic model is an effective method for the automatic organization and induction of text information. It can reveal the latent semantics of documents and analyze the topics contained in massive information. Therefore, this paper proposes a multi-label text classification method based on tALBERT-CNN: an LDA topic model and ALBERT model are used to obtain the topic vector and semantic context vector of each word (document), a certain fusion mechanism is adopted to obtain in-depth topic and semantic representations of the document, and the multi-label features of the text are extracted through the TextCNN model to train a multi-label classifier. The experimental results obtained on standard datasets show that the proposed method can extract multi-label features from documents, and its performance is better than that of the existing state-of-the-art multi-label text classification algorithms.

## Introduction

Automatic text classification is an important means for humans to process massive amounts of text information. In the real world, due to complex and changeable text data environments and the existence of polysemous objects, text classification face many severe challenges. The traditional single-label text classification method has not fully met the needs of users. To better meet the needs of users for text classification tasks, the multi-label learning method came into being [1]. Multi-label learning refers to the process of assigning the most relevant subset of class labels to each instance from the overall label set, thereby intuitively reflecting the various semantic information contents of ambiguous objects. For example, a news report about coronavirus disease 2019 (“COVID-19”) is likely to belong to the “fighting epidemic” category, the “medical and health” category, and the “economic crisis” or “national security” category.

Multi-label text classification is one of the important branches of multi-label learning, and it is mainly used in sentiment analysis, topic labeling, question answering, and dialog behavior classification [2–5]. Multi-label text data have the following characteristics. Multi-label text classification allows a document to belong to multiple labels, so the different levels and aspects of semantic features need to be captured; documents may be relatively long, and complex semantic information may be hidden in noisy or redundant content; most documents belong to only a few labels, and a large number of “tail labels” have only a few training documents [6]. Due to the characteristics of multi-label text data, researchers mainly focus on three aspects: how to accurately mine the correlation between labels; how to accurately represent the complex semantics of the given documents, especially through the use of domain knowledge to supplement the semantic information of the document; and how to fully capture the effective information from each document and extract the feature information related to the corresponding label. The emergence of attention mechanisms, combined with deep neural networks, can effectively solve the problem of long-distance word dependencies, and capture important words in the document. In particular, in 2017, Vaswani et al. [7] proposed a new transformer network structure in the paper titled “Attention Is All You Need”; this structure is not only faster than other approaches during training, but is also more suitable for modeling long-distance dependencies. It has achieved very good results on many NLP tasks. Since then, an increasing number of institutions and scholars have conducted extensive research based on transformers and produced many excellent language models, such as the OpenAI GPT and BERT. These excellent language models have been widely used in multi-label learning tasks. However, these models generally have large numbers of parameters and express only the local semantics accurately, so they cannot represent the macrosemantic information of documents. In 2020, Lan et al. [8] proposed “A Lite BERT” (ALBERT) model, which greatly simplifies the number of required parameters. Peinelt et al. [9] combined a topic model with a BERT model for the task of semantic similarity detection. We have reason to use ALBERT and topic models to extract important information of different granularities form documents to further improve the effect of multi-label text classification. Using deep learning methods to solve multi-label problems, the purpose is to find the mapping relationship between text features and labels. At present, this mapping relationship is not very clear. Therefore, we will attempt to use different levels and granularity of features (e.g., semantic information and topic information) to represent the depth features^1^ of the text, and map them to the label space.

Through the above analysis, although multi-label learning has received extensive attention and made much progress, some problems and challenges still must be further studied and solved. Among them, how to combine topic information and semantic information to guide multi-label text classification is the key problem. Therefore, this paper proposes a depth semantic model that integrates the topic information of the document domain and the local contextual semantic information of the input document to obtain the depth feature representation of the document; then, a convolution neural network (CNN) is used to extract depth features at different levels. In this paper, a latent Dirichlet allocation (LDA) topic model is used to obtain word-level and document-level topic information, and a certain fusion mechanism is used to represent the topic and semantic depths of the document. Then, the depth feature of the document is extracted by the CNN model, and the probability of each label is calculated by a fully connected network (FCN) and a sigmoid function. Finally, the cross-entropy loss function is used for training.

Our contributions can be summarized as follows:

1. In this paper, we propose a method called topic ALBERT (tALBERT), which combines an LDA topic model and the ALBERT model to represent the depth features of documents.
2. We design a multi-label text classification model based on tALBERT and TextCNN. The combined model can obtain different levels of semantic document information, extract the depth features^2^ of documents, and improve the prediction effect of the model.
3. This paper evaluates the performance of the proposed method and compares it with the current representative multi-label text classification methods using three benchmark datasets. The experimental results show that the proposed method is better than the baseline models.

## Related Works

With the rapid development of machine learning, especially deep learning, many classification methods have been proposed to solve the multi-label learning problem. These methods mainly focus on research of with traditional machine learning algorithms and deep learning models. Among them, traditional machine learning methods include problem transformation methods and algorithm adaptation methods; deep learning methods are mainly divided into CNN-based, RNN-based, and transformer-based multi-label text classification methods according to their model structures.

### Traditional Machine Learning Methods

According to different solution strategies, traditional machine learning methods can be divided into two categories: problem transformation methods and algorithm adaptation methods [10].

Problem transformation methods: This category of algorithms tackles multi-label learning problems by transforming them into single-label learning tasks. Representative algorithms include first-order approaches, second-order approaches, and high-order approaches. Binary relevance (BR) [11] is the most representative first-order problem transformation method. The basic idea of this algorithm is to decompose a multi-label learning problem into several independent binary classification problems. However, due to its in ability to discover the interdependence between labels, BR may lead to a decrease in prediction performance. The typical algorithm among the second-order approaches is calibrated label ranking (CLR) [12]. The basic idea of the CLR algorithm is to transform a multi-label learning problem into a label ranking problem and use pairwise comparison technology to realize the rankings between labels. Although CLR has the advantage of reducing the imbalance between label categories, the number of binary classifiers constructed by CLR increases from a linear value to a square value as the number of labels changes. Therefore, this method has limitations and is not suitable for sample data with a large number of labels. Classifier chains (CCs) [13] and label powersets (LPs) [1] are typical high-order problem transformation methods. A CC is an improvement of the BR method that does not consider the correlations between labels and leads to the loss of information. The basic idea of the CC algorithm is to transform a multi-label learning problem into a series of binary classification problems, in which the subsequent binary classifiers in the chain are based on the prediction of the previous classifier. Therefore, when the previous label predicts an error, the error is passed down the chain. The basic idea used by the LP algorithm to solve problems is to transform a multi-label learning problem into a set of multi-class classification problems. Each subset generates a new set of labels via LP technology, and a multi-class label is finally learned for each subset. However, this method may result in sample imbalance after the initial problem is transformed. In other words, with increases in the number of labels and the sample space, these methods face great challenges in terms of computational efficiency and performance.

Algorithm adaptation methods: This category of algorithms tackles multi-label learning problems by adapting popular learning techniques to deal with multi-label data directly. These techniques mainly include first-order approaches and second-order approaches. Multi-label k-Nearest Neighbors (ML-kNN) [14] and Multi-label Decision Trees (ML-DTs) [15] are typical first-order approaches. The basic idea of the ML-kNN algorithm is to adapt k-nearest neighbor techniques to deal with multi-label data, where the maximum a posteriori (MAP) rule is utilized to make predictions by reasoning with the labeling information embodied in neighbors. The ML-kNN algorithm can mitigate the class-imbalance issue by estimating the prior probability of each class label, but the computational complexity of this approach is high. The basic idea of the ML-DT algorithm is to adopt decision tree techniques to deal with multi-label data, where an information gain criterion based on multi-label entropy is utilized to build the decision tree recursively; however, the algorithm assumes that the labels are independent when calculating the multi-label entropy. Typical second-order approaches include Ranking Support Vector Machine (Rank-SVMs) [16] and Collective Multi-Label Classifier (CMLs) [17]. The basic idea of the Rank-SVM algorithm is to adapt a maximum margin strategy to deal with multi-label data, where a set of linear classifiers is optimized to minimize the empirical ranking loss and enabled to handle nonlinear cases with kernel tricks. Rank-SVM is a machine learning algorithm based on statistical learning theory that extends the classical SVM to multi-label learning problem. The basic idea of the CML algorithm is to adapt the maximum entropy principle to deal with multi-label data, where the correlations among labels are encoded as constraints that the resulting distribution must satisfy. The CML algorithm takes the correlation between labels into account, but the complexity of the algorithm is too high.

### Deep Learning Methods

With the development of deep neural networks, researchers have proposed a variety of deep learning methods for multi-label text classification, including CNNs, RNNs, and transformer-based deep neural network models. In 2014, Kim et al. [18] proposed the TextCNN model, which first uses a CNN structure for text classification and then uses a CNN for sentence-level classification; the authors carried out a series of experiments based on Word2vec word embeddings. However, this model cannot avoid the disadvantage of utilizing fixed windows in CNNs, so it cannot model long sequence information. Liu et al. [19] improved the structure of TextCNN and proposed an XML-CNN model. This model is different from TextCNN in that dynamic pooling is used in the pooling operation, the loss function is improved, the binary-cross-entropy loss function is adopted, and a hidden layer is added between the pooling layer and output layer; this layer can map high-dimensional labels to low-dimensional to reduce the number of required calculations. Yang et al. [20] proposed a twin hyperspectral CNN (HSCNN) for multi-label text classification with unbalanced data. This network mainly deals with small-sample problem with the twin network structure and uses a hybrid mechanism to solve extremely unbalanced multi-label text classifications. The head label adopts a single network structure, and the tail label adopts a twin network with less sampling. A multi-label text classification method based on a CNN is relatively simple and does not need to incur a massive computational cost. However, the pooling operation of a CNN causes the loss of semantic information, and when the text is too long, a CNN is not conducive to capturing the relationship between the preceding and the following information, resulting in semantic deviation.

Nam et al. [21] used an RNN to replace the classifier chain in a CNN and used a sequence-to-sequence (seq2seq) based on an RNN to perform modeling. The method can generate label sequences in turn by RNN to capture the correlation between labels. This was the first time that the seq2seq model was applied to multi-label text classification. After that, more seq2seq models were proposed to deal with multi-label text classification. Chen et al. [22] proposed a fusion mechanism for a CNN and an RNN. First, a word vector is sent to the CNN to obtain the corresponding text feature sequence, and then, the feature is input into the RNN to obtain the corresponding prediction label. However, the model is greatly influenced by the size of the given training set. If the training set is too small, overfitting may result. Most multi-label text classification methods based on RNNs are implemented using the seq2seq structure, which considers the relationships between labels using sequence generation. The latter label is often dependent on the former label, so the impact of incorrect labels is often superimposed. Although some methods have been improved in this regard, some defects remain. This improvement improves the model effect to some extent, but whether the model can effectively learn the correlations between well remains to be discussed.

The typical network structure of a transformer adopts an attention mechanism; this is unlike the traditional encoder–decoder model, which needs to be combined with an RNN or a CNN. The proposal of the transformer has greatly influenced the field of NLP, especially the proposal of the BERT model based on a transformer structure, which is said to be a milestone of NLP. Yarullin et al. [23] first tried BERT and explored it under multi-label settings and in hierarchical text classification problems and proposed a sequence-generating BERT model in the field of multi-label text classification. Chang et al. [24] proposed the X-Transformer model, which is composed of three parts, including a semantic label sequence component, a deep neural matching component, and an overall ranking component. Gong et al. [25] proposed the deep learning model of HG-transformer, which first models the input text as a graph structure; then uses a multi-layer transformer structure with a multi-attention mechanism at the word, sentence, and graph levels to fully capture the characteristics of the text; and finally utilizes the hierarchical relationships among the labels to generate t label representations. A weighted loss function was designed based on the semantic distances among labels. The effect of a multi-label text classification model based on a transformer structure is often better than that of models based on CNN and RNN structures, but the number of model parameters required for a transformer model is often large, and the network structure is complex, producing some limitations in practical application.

To further improve the applicability and performance of multi-label text classification in real scenarios, this paper proposes a joint model called tALBERT, which combines LDA and ALBERT, to obtain different multi-level document representations. On this basis, TextCNN is used to extract the depth features of documents and to conduct multi-label text classification.

## tALBERT-CNN Method

This section mainly introduces our multi-label text classification method called tALBERT-CNN, primarily including a description of the multi-label classification problem, the model framework, topic information extraction based on LDA, text representation based on tALBERT, multi-label learning, and prediction.

### Problem Description

Assume that $X={\mathbb{R}}^{d}$ represents the *d*-dimensional feature vector input space of the instance and that $Y=\{{y}_{1},{y}_{2},\dots ,{y}_{q}\}$ represents the *q*-dimensional label output space of the instance. Then, the dataset for multi-label learning can be defined as $D=\{({x}_{i},{Y}_{i})|1\le i\le N\}$, where *N* is the number of document, ${x}_{i}\in X$ is the *d*-dimensional feature vector of the instance, and ${Y}_{i}\subseteq Y$ is the label set corresponding to instance ${x}_{i}$. In this way, the multi-label learning task can actually be transformed into finding a suitable mapping function $h: X\to {2}^{y}$ from the training set, so that the input space of the feature vector can be mapped to the output space of the label set through this mapping function. When instance x with an unknown label is reached, the label set can be predicted through the mapping function $h(x)\subseteq Y$. Commonly, when the feature vector *x* of an instance is given, we can use the learning function to obtain a set of 0/1 vectors about the label space. When ${Y}_{i}\subseteq Y$,${Y}_{i}=1;$ otherwise, ${Y}_{i}=0$.

### Model Framework

**Fig. 1:**
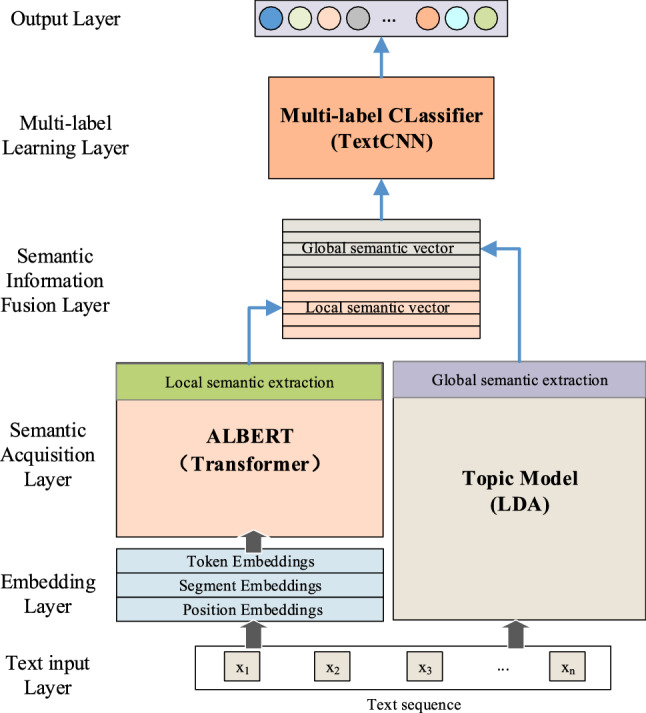
Multi-label learning framework based on tALBERT-CNN

In this section, the method proposed in this paper is introduced in detail. Due to the large number of parameters required by the BERT model and its advantages in local semantic representation, this model cannot represent the macro-domain information of the input document other than its own semantics. Inspired by reference [8] and reference [9], we propose a document semantic acquisition method based on tALBERT. We obtain the topic information at the word level and document level through an LDA topic model, obtain a semantic representation at the word-level and document-level through ALBERT, and fuse the above information through a concatenation mechanism to represent the document. Compared with other attention mechanisms, a CNN has the characteristic of efficiently capturing features between different words, so we choose TextCNN as the multi-label feature extraction model for multi-label learning and classification prediction. The model frame is shown in Fig. 1.

In addition, before the text sequence is input into the multi-label classification framework (Fig. 1), need to do the following work:

1. Document preprocessing. It mainly includes removing invalid symbols, digital normalization, converting all uppercase English characters to lowercase and lemmatization, etc. the text sequence set is formed after preprocessing.
2. Training LDA model.
3. Fine-tune the ALBERT model. If the length of the text sequence is greater than 512 words, it is truncated and then sent to the ALBERT model.

### Topic Information Extraction

**Fig. 2:**
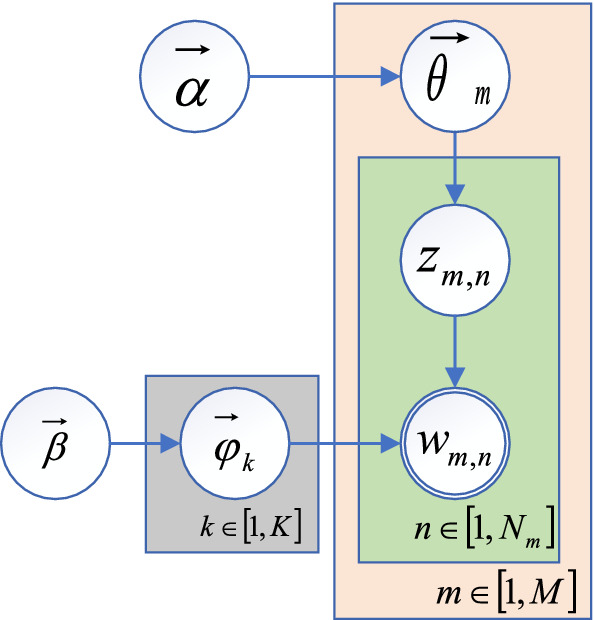
LDA topic model

A topic model was the first developed text analysis tool and is a popular language model. It is an effective unsupervised tool that can reveal the latent semantic information in the input text corpus based on the global text context information of the corpus. Topic models refer to probabilistic latent semantic analysis (PLSA), LDA, and various extensions. Among them, LDA is the most complete probabilistic topic model. An LDA topic model is a feature extraction method based on the Bag of Words (BOW) model. It ignores the order information of words and the information between context words. It consists of three levels of probability distributions: document, topic, and word levels. The topic information is added to the document-word feature level, the word information is mapped to the topic space, and the global underlying semantic structure of the text is captured to achieve a good representation of the text features in the hidden topic space. An LDA topic model directly captures the global semantics related to words in the text and obtains a global feature representation of the text. Figure 2 shows the detailed process of the LDA model for generating topic information.

1. For any topic *z*, obtain the polynomial distribution of the words under this topic according to the Dirichlet distribution ${\varphi }_{k}$, i.e., ${\varphi }_{k}\sim \mathrm{Dirichlet}(\beta )$, where $\beta$ is an a priori hyperparameter that is generally set to 0.01.
2. For each document ${w}_{m}$, its topic probability ${\theta }_{m}$ obeys a Dirichlet distribution, which is ${\theta }_{m}\sim \mathrm{Dirichlet}(\alpha )$, where $\alpha$ is an a priori hyperparameter that is generally set as $50/K$ and *K* is the number of topics.
3. For each document ${w}_{m}$ in the training corpus and all vocabulary ${w}_{m,n}$ in the document, traverse: choose topics ${z}_{m,n}$ and ${w}_{m,n}$; they all obey multinomial distributions, which are ${z}_{m,n}\sim \mathrm{Multinomial}\left({\theta }_{m}\right), {w}_{m,n}\sim \mathrm{Multinomial}({\varphi }_{k})$.

Based on reference [26], in which word-level and document-level topics were successfully combined with a neural architecture, we can easily obtain the topic ${Z}_{i}$ of each document, and all tags in a document are passed to the topic model to infer each document theme distribution; see Eq. (1)

1 $${Z}_{i}=\mathrm{LDA}\left(\left[{T}_{1},\dots ,{T}_{N}\right]\right)\in {R}^{k},$$

where $i$ denotes the number of document and $K$ denotes the number of topics. In addition, for a word-level topic $W$, a topic distribution ${w}_{j}$ is inferred from each tag ${T}_{i}$. See Eq. (2)

2 $${w}_{j}=\mathrm{LDA}\left({T}_{i}\right)\in {R}^{k}.$$

### Text Representation Based on tALBERT

BERT as a replacement for Word2vec has greatly improved its accuracy in 11 directions of the NLP field. A BERT model has the following three characteristics. By utilizing a transformer as the main framework of the algorithm, the bidirectional relationships in sentences can be more thoroughly captured. The algorithm uses a mask language model (MLM) [27] and next sentence prediction (NSP) as the goal of multi-task training. Large-scale training data have enabled the results of BERT reach new heights, and Google has made their BERT model open source. Researchers can directly use BERT as the conversion matrix of Word2vec and efficiently apply it to their own tasks. Although BERT has many advantages, the basic version of the BERT model possesses as many as 110 M parameters, and the GPU memory utilization is as greater as 7 GB during the training process. A large BERT model has as many as 340 M parameters, and the GPU memory occupied by the training process is as high as 32 GB, which is a problem for researchers. Therefore, ALBERT has emerged to fill this need. Compared with BERT, ALBERT is mainly improved in terms of two aspects to reduce the number of parameters.

#### Factorized Embedding Parameterization

For BERT, the word vector dimensionality *E* and the hidden layer dimensionality *H* are equal. With the increase in the dimensions of the model (that is, as the word vector dimensionality and hidden layer dimensionality increase), the parameter quantity of the model increases rapidly. Reference [8] provided a method to decompose parameters: in the mapping process from the vocabulary dimensionality *V* in the input layer to the hidden layer dimensionality *H*, *V* is first projected into a low-dimensional embedded space *E*, and then, E is projected into hidden layer *H* (usually, the dimensionality of *H* is much larger than that of *E*), and the number of parameters after completing a transformation is $O\left(V\times H\right) \mathrm{to} O\left(V\times E+E\times H\right),$ thereby reducing the number of embedding parameters.

#### Cross-Layer Parameter Sharing

The author of the paper that introduced ALBERT proposed cross-layer parameter sharing as another method to improve parameter efficiency. There are four ways to share parameters, namely, attention parameter sharing, feed-forward network (FFN) parameter sharing, cross-layer parameter sharing, and no sharing. Table 1 compares the configurations of the BERT and ALBERT models. Table 2 compares the parameters of different cross-layer sharing methods with the base ALBERT.

**Table 1:** BERT and ALBERT model configuration comparison

Model		Parameters	Layers	Hidden	Embedding	Parameter sharing
**BERT**	**Base**	108 M	12	768	768	False
	**Large**	334 M	24	1024	1024	False
**ALBERT** ***E* = 128**	**Base**	12 M	12	768	128	True
	**Large**	18 M	24	1024	128	True

**Table 2:** Parameters of the base ALBERT

Model (ALBERT base)	Cross-layer parameter sharing strategies			
	All-shared	Shared attention	Shared-FFN	Not shared
***E* = 128**	12 M	64 M	38 M	89 M
***E* = 768**	31 M	83 M	57 M	108 M

Based on the full experiment of ALBERT in reference [8], we use the ALBERT model and adopt a single-sentence (document) input mode to obtain word-level (document-level) semantic representations.

1. Document-level semantic vector acquisition: Obtain the semantic vector ${C}_{i}$ of each document ${D}_{i}$ (length less than 512 words) from the *CLS* tag of the last layer of ALBERT, and see Eq. (3)
  3 $${C}_{i}=\mathrm{ALBERT}\left({D}_{i}\right)\in {R}^{d}.$$
2. Word-level semantic vector acquisition: For each document ${D}_{i}$, obtain the semantic representation vector ${\mathrm{v}}_{\mathrm{ij}}$ of each word from the token of the last layer of ALBERT; and see Eq. (4)
  4 $${v}_{ij}=\mathrm{ALBERT}\left({D}_{i}\right)\in {R}^{d},$$
  where *d* denotes the internal hidden size of ALBERT (768 for base ALBERT or1024 for large ALBERT). Based on formulas (1)–(4), we use two fusion methods and three specific strategies for document feature representation.
3. Document-level information fusion strategy ${S}_{1}$: concatenate the document-level topic vector ${Z}_{i}$ and semantic vector ${C}_{i}$ by row, and see Eq. (5)
  5 $${S}_{1}=\left[{Z}_{i};{C}_{i}\right]\in {R}^{k+d}.$$
4. Word-level information fusion strategy ${\mathrm{S}}_{2}$: Concatenate the topic vector ${\mathrm{w}}_{\mathrm{i}}$ and word vector ${\mathrm{v}}_{\mathrm{i}}$ of each word in the document by column. The dimensions of the two types of vectors should be the same, i.e., $k=d$; see Eq. (6)
  6 $${S}_{2}=\left[ \begin{array}{c}{w}_{1} \\ {w}_{2} \\ \begin{array}{c}\dots \\ {w}_{l} \\ \begin{array}{c}{v}_{1} \\ {v}_{2} \\ \begin{array}{c}\dots \\ {v}_{l} \end{array}\end{array}\end{array}\end{array}\right]\in {R}^{2l\times d}.$$
5. Hybrid information fusion strategy ${S}_{3}$: Concatenate the topic vector ${w}_{i}$ of each word, the document-level topic vector ${Z}_{i}$, the document-level semantic vector ${C}_{i}$, and the word vector ${v}_{i}$ by column. The dimensions of the four types of vectors should be consistent, namely, $k=d$; see Eq. (7)
  7 $${S}_{3}=\left[ \begin{array}{c}{w}_{1} \\ {w}_{2} \\ \begin{array}{c}\dots \\ \begin{array}{c}{w}_{l}\\ \begin{array}{c}{Z}_{i}\\ {C}_{i}\end{array}\end{array} \\ \begin{array}{c}{v}_{1} \\ {v}_{2} \\ \begin{array}{c}\dots \\ {v}_{l} \end{array}\end{array}\end{array}\end{array}\right]\in {R}^{2\left(l+1\right)\times d}.$$

Among them, ${S}_{1}$ is used for the input of the fully connected layer and is finally used for multi-label classification prediction, and ${S}_{2}, {S}_{3}$ are used as the inputs of TextCNN to extract multi-label features at different levels for multi-label classification and prediction.

### Multi-label Learning and Prediction

**Fig. 3:**
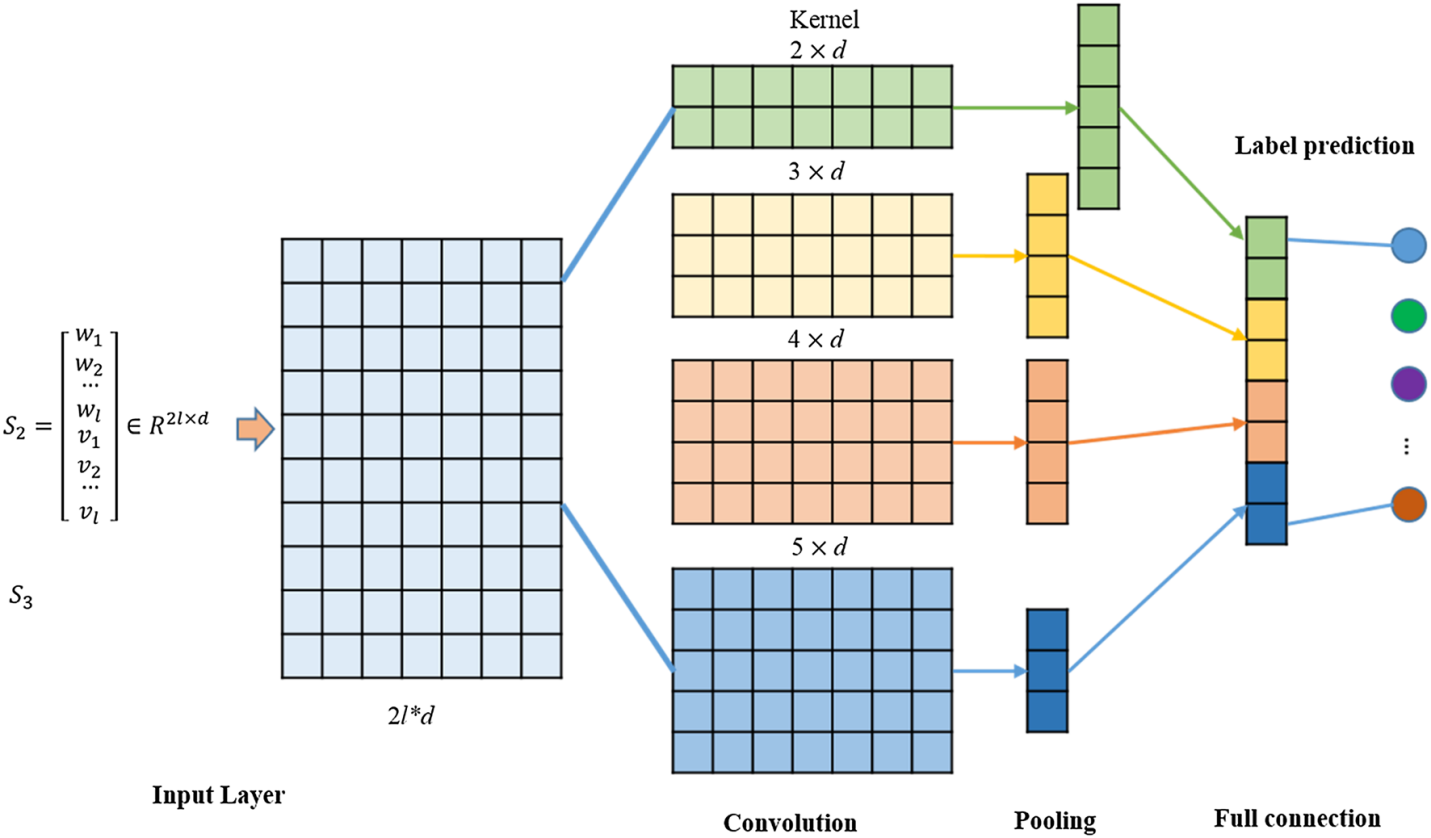
Multi-label classification model based on TextCNN

In this section, we mainly introduce the proposed multi-label prediction model based on TextCNN. The specific model structure is shown in Fig. 3. The training model consists of an embedding layer, a convolutional layer, a pooling layer, and a fully connected layer.

#### Embedding Layer

Each document and word in the dataset can be represented by a semantic feature vector. The semantic feature vector acquisition method adopted in this paper combines a dynamic semantic vector and a static topic vector. Through the ALBERT language model, the semantic vector of the document and the contextual semantic vector of each word in the document can be obtained to solve ambiguity problem; the document-level topic vector and the topic vector of each word can be obtained through the LDA topic model. These topic vectors largely imply the domain knowledge in which the document or word is located. To meet the data format requirements of the embedding layer, we uniformly set the document length to *L* words. For documents longer than *L*, we intercept the first *L* words, and for words whose lengths are less than w, we fill the remainder of the document with 0 s. Based on the research in the previous section, we set the dimensionality *d* of the topic vector and the word vector to 768 and set the fusion strategies ${S}_{2}\in {R}^{2L\times ,768}$, ${S}_{3}\,{\in R}^{2(L+1)\times 768}$ as for the embedding vector.

#### Convolutional Layer

The convolutional layer is used to extract the different pieces of granular feature information contained in the semantic feature vector. This task can be achieved by setting convolution kernels with different size. The width of the convolution kernels defined in this paper is the word vector dimensionality *d*. According to different languages, different heights *h* can be selected for the convolution kernels. Using more convolution kernels with different heights, a richer feature representation can be obtained (in this paper, the heights *h* of the convolution kernels are set to 2, 3, 4, and 5).

#### Pooling Layer

The pooling layer reduces the output result of the convolutional layer and extracts a deeper feature representation. The sizes of the feature sets obtained by the convolution kernels with different heights are different. This paper uses the pooling function for each feature set and uses max pooling to extract the maximum value in the feature collection. For each convolution kernel, the output feature is the maximum value of the feature set, max pooling is used for all convolution kernels, all output feature values are concatenated, and the final feature vector representation of the document is obtained.

#### Fully Connected Layer and Loss Function

The feature vector obtained after concatenating the output result of the pooling layer is fully connected with *q* neurons (the same as the number of label sets) as the output layer of the model. At the same time, the *sigmoid* function is used as the output function of the model, and its formula is

8 $${p}_{ij}=\mathrm{sigmoid}\left(x\right)=\frac{1}{1+{e}^{-x}}.$$

Finally, to determine whether the document belongs to the given label, this paper sets the threshold to 0.5; that is, ${p}_{ij}\ge 0.5$ means that this label is used as one of the output labels of the current instance; otherwise, it is not used as the output label of the current instance.

In addition, this paper uses the *cross-entropy* function as the loss function for training the model. The formula is

9 $${L}_{\mathrm{loss}}=-\sum_{i=1}^{N}\sum_{j=1}^{q}[{y}_{ij}\times \mathrm{ln}{p}_{ij}+(1-{y}_{ij})\times \mathrm{ln}{(1-p}_{ij})],$$

where *N* denotes the number of documents and *q* is the number of labels; ${p}_{ij}\in \left[0,1\right], {y}_{ij}\in \{0,1\}$, which are the predicted value and true value of the *j*th label of the *i*th instance, respectively.

## Experiments

To prove the effectiveness of the multi-label text classification method proposed in this paper, in this section, we provide a discussion in four parts: a description of the datasets, the selection of the evaluation metrics, comparisons among various methods and parameter settings, and a comparison of experimental results.

### Datasets

In our paper, we use the following three multi-label text classification datasets: the arXiv Academic Paper Dataset (AAPD), the Internet Movie Database (IMDB), and Reuters Corpus Volume I (RCV1).

The AAPD collects the abstracts and the corresponding subjects of 55,840 papers in the field of computer science on the arXiv website. Each paper may involve multiple subjects (labels) (for a total of 54 subjects), and each abstract has one or more subject marks. Since the text content includes academic papers, the text is relatively standardized, and the label settings are relatively reasonable. The model can predict the corresponding subject of the paper based on the abstract content, making the AAPD very suitable as a dataset for multi-label text classification models and algorithm research.

The IMDB contains 117,196 movie introductions (in English), with a total of 27 movie categories. Each movie introduction has one or more possible types. The dataset provides a multi-label binary mask for each movie according to whether the movie belongs to a specific type. Therefore, this dataset is suitable for multi-label classification models and algorithm research.

RCV1 has a total of 804,414 news reports, involving 103 categories. Each report may contain one or more categories. On average, each news report contains 3.2 category labels. The data can be used to test the performance of the method proposed in this paper in a case with a large-scale dataset and a large number of labels.

Table 3 lists the statistics of these datasets, where *N* is the number of total instances, $\overline{W }$ is the average number of words per document in the dataset, $Q$ is the total number of classes, and $\overline{Q }$ is the average number of labels per document.

**Table 3:** Overview of the experimental datasets

Datasets	*N*	$\overline{W }$	$Q$	$\overline{Q }$
**AAPD**	55,840	163.4	54	2.4
**IMDB**	117,196	98.4	27	2.2
**RCV1**	804,414	268.9	103	3.2

Since the lengths (number of words) of the documents in the original datasets are different, a document that is too short will result in the inability to accurately determine the category of the text, and a document that is too long will result in a waste of space. According to the characteristics of each dataset, this paper fits the document lengths of the AAPD, IMDB, and RCV1 datasets to 250,150, and 300 for the input of the model. If the document length exceeds the set value, we cut it off, and if the length is insufficient, we fill it with 0 s. In addition, if the length of the document is less than 20 words, the document is directly discarded in the paper.

### Evaluation Metrics

To comprehensively evaluate the method proposed in this paper, we choose commonly used sample-based evaluation metrics. The effectiveness of the method is mainly evaluated by its precision (P), recall (R), F1 score (F1), subset accuracy (SA), and Hamming loss (HL), and these scores are compared with those of other methods.

*P*: precision reflects the average of the percentage of correctly predicted labels and predicted labels in all samples.

10 $$P=\frac{1}{\left|N\right|}\sum_{i=1}^{\left|N\right|}\frac{\left|\widehat{{y}_{i}}\cap {y}_{i}\right|}{\left|\widehat{{y}_{i}}\right|},$$

where $\left|N\right|$ denotes the total number of test samples.

*R*: recall reflects the average of the percentages of correctly predicted labels and true labels in all samples.

11 $$R=\frac{1}{\left|N\right|}\sum_{i=1}^{\left|N\right|}\frac{\left|\widehat{{y}_{i}}\cap {y}_{i}\right|}{\left|{y}_{i}\right|}.$$

*F*1: the F1 Score is a comprehensive metric that combines precision and recall. The larger the value, the better the system performance.

12 $$F1=\frac{2P\times R}{P+R}.$$

SA: subset accuracy evaluates the fraction of correctly classified examples, i.e., whether the predicted label set is identical to the ground-truth label set. Intuitively, subset accuracy can be regarded as a multi-label counterpart of the traditional accuracy metric and tends to be overly strict especially when the size of the label space is large.

13 $$SA=\frac{1}{\left|N\right|}\sum_{i=1}^{\left|N\right|}1\left\{\widehat{{y}_{i}}={y}_{i}\right\},$$

where $1\{\widehat{{y}_{i}}={y}_{i}\}$ means that if the label is true 1 is returned; otherwise, 0 is returned.

HL: The Hamming loss measures the proportion of misclassified labels, the proportion of labels whose correct labels are not predicted, and the proportion of labels whose incorrect labels are predicted. The smaller the value of the Hamming loss is, the more effective the tested model or method.

14 $$HL=\frac{1}{\left|N\right|}\sum_{i=1}^{\left|N\right|}\frac{\mathrm{xor}(\widehat{{y}_{i}},{y}_{i})}{q},$$

where *q* denotes the total number of labels, $\widehat{{y}_{i}}$ and ${y}_{i}$ denote the predicted label and the real label, respectively, and xor denotes the XOR operation.

### Comparison Method and Parameter Setting

The multi-label text classification method proposed in this paper is fundamentally composed of two parts: deep topic and semantic representation based on tALBERT and multi-label feature learning based on a CNN. Therefore, the chosen baseline models also adopt similar network structures. In addition, to fully verify the performance of the method in this paper, we also choose other excellent models based on RNNs and attention mechanisms for comparison purposes.

#### Comparison Method

TextCNN [18]: This method is based on Word2vec for word embedding, and for the first time, a CNN structure is used for text classification.

XML-CNN [19]: Using a CNN to design a dynamic pool to deal with text classification, this method is a representative algorithm for processing text classification task.

DTFEM-ML_KNN [28]: This method uses a combination of LDA and bidirectional long short-term memory (Bi-LSTM) to extract deep document topic features and is combined with the traditional machine learning method ML_KNN for multi-label text classification.

Label-Specific Attention Network (LSAN) [6]: The algorithm uses an adaptive fusion strategy to obtain document representations via a self-attention mechanism and a label attention mechanism, and finally combines the two types of document representations to construct a multi-label text classifier.

#### Our Method

According to the tALBERT document feature representation method and the three different information fusion strategies proposed in this paper for different levels (${S}_{1}$,${S}_{2}$ and ${S}_{3}$), the tested multi-label text classification methods mainly include tALBERT-S1, tALBERT-CNN-S2, and tALBERT-CNN-S3.

#### Parameter Setting

For TextCNN and XML-CNN, we use Google’s pre-trained Word2vec as the word embedding mechanism, the embedding dimensionality $d=300$, the convolution kernel width is set to 300, and the heights are set to $\{\mathrm{2,3},\mathrm{4,5}\}$.

We set DTFEM-ML_KNN and the LSAN according to the parameters in their original papers.

The proposed method selects the pre-training model under the base ALBERT ($d=768$) in the all sharing mode with $E=128$ (see Table 1). For the LDA topic model, we set the number of topics $k=128$, and the hyperparameters $\alpha =0.5$ and $\beta =0.01$. The widths of the convolution kernels are set to 768, and the heights are set to $\{\mathrm{2,3},\mathrm{4,5}\}$.

### Experimental Results

In this section, the proposed tALBERT-CNN is evaluated on three benchmark datasets via a comparison with five baselines in terms of P, R, F1, SA, and HL. Tables 4, 5, and 6 show the performance of the LDA, ALBERT, and tALBERT-CNN models with different fusion strategies for all test documents. The LDA and ALBERT models adopt document-level vector representation, an FCN, and a sigmoid function to achieve multi-label text classification. Tables 7, 8, and 9 show the comparison of our proposed model with other baseline models on the three datasets. “** + **” denotes that the larger the value is, the better the model performance, and “**−**” represents that the smaller the value is, the better the model performance. In each line, the best result is marked in bold.

**Table 4:** Experimental results obtained by the tALBERT-CNN model with different fusion strategies (AAPD)

Models	*P*( +)	*R*( +)	*F*1( +)	SA( +)	HL(−)
**LDA**	0.7372	0.5068	0.6043	0.2869	0.0328
**ALBERT**	0.7265	0.6068	0.6643	0.3567	0.0298
**tALBERT- S1**	0.7446	0.6571	0.7039	0.3576	0.0291
**tALBERT-CNN-S2**	0.7513	0.6589	0.7143	0.3735	0.0285
**tALBERT-CNN-S3**	**0.7519**	**0.6695**	**0.7317**	**0.3806**	**0.0276**

Best results are marked in bold

**Table 5:** Experimental results obtained by the tALBERT-CNN model with different fusion strategies (IMDB)

Models	*P*( +)	*R*( +)	*F*1( +)	SA( +)	HL(−)
**LDA-FCN**	0.6587	0.4266	0.5219	0.2678	0.0704
**ALBERT-FCN**	0.7736	0.6456	0.7042	0.4248	0.0404
**tALBERT- S1**	0.8044	0.7256	0.7689	0.4653	0.0336
**tALBERT-CNN-S2**	0.8247	0.7396	0.7999	0.4748	0.0309
**tALBERT-CNN-S3**	**0.8431**	**0.7585**	**0.8023**	**0.4825**	**0.0286**

Best results are marked in bold

**Table 6:** Experimental results obtained by the tALBERT-CNN model with different fusion strategies (RCV1)

Models	*P*( +)	*R*( +)	*F*1( +)	SA( +)	HL(−)
**LDA-FCN**	0.8223	0.7355	0.7836	0.3218	0.0308
**ALBERT-FCN**	0.8548	0.8337	0.8421	0.4718	0.0288
**tALBERT- S1**	0.8716	0.8344	0.8519	0.4776	0.0257
**tALBERT-CNN-S2**	0.8805	0.8523	0.8681	0.4829	0.0237
**tALBERT-CNN-S3**	**0.8932**	**0.8559**	**0.8792**	**0.4868**	**0.0233**

Best results are marked in bold

**Table 7:** Experimental results of different models (AAPD)

Models	*P*( +)	*R*( +)	*F*1( +)	SA( +)	HL(−)
**TextCNN**	0.6312	0.5506	0.5732	0.2998	0.0458
**XML-CNN**	0.6581	0.5884	0.6235	0.3021	0.0410
**DTFEM-ML_KNN**	0.7072	0.5738	0.6335	0.3276	0.0295
**LSAN**	0.7325	0.6517	0.7019	0.3787	0.0289
**tALBERT-CNN-S3**	**0.7519**	**0.6695**	**0.7317**	**0.3806**	**0.0276**

Best results are marked in bold

**Table 8:** Experimental results of different models (IMDB)

Models	*P*( +)	*R*( +)	*F*1( +)	SA( +)	HL(−)
**TextCNN**	0.7195	0.6152	0.6714	0.4015	0.0437
**XML-CNN**	0.7287	0.6475	0.6905	0.4234	0.0434
**DTFEM-ML_KNN**	0.7712	0.6593	0.7108	0.4773	0.0429
**LSAN**	0.8317	0.7426	0.7952	0.4785	0.0325
**tALBERT-CNN-S3**	**0.8431**	**0.7585**	**0.8023**	**0.4825**	**0.0286**

Best results are marked in bold

**Table 9:** Experimental results of different models (RCV1)

Models	*P*( +)	*R*( +)	*F*1( +)	SA( +)	HL(−)
**TextCNN**	0.7936	0.7625	0.7804	0.4571	0.0352
**XML-CNN**	0.8259	0.7738	0.7913	0.4619	0.0341
**DTFEM-ML_KNN**	0.8704	0.8415	0.8574	0.4801	0.0245
**LSAN**	**0.9016**	0.8507	**0.8823**	0.4836	0.0235
**tALBERT-CNN-S3**	0.8932	**0.8559**	0.8792	**0.4868**	**0.0233**

Best results are marked in bold

From Tables 4, 5, 6, we can find that our method is obviously superior to the LDA topic model due to its use of probability feature statistics and the deep semantic model ALBERT. This fully shows that the combination of a topic model and deep semantic model significantly improves NLP downstream tasks performance, which is consistent with the conclusion of reference [9]. In addition, the effects of different fusion methods on multi-label text classification are also different. The effect of only fusing document-level topic vectors and semantic vectors is the worst, but the results are better than those of a single model. The effect of fusing word-level and document-level semantic vectors is optimal for multi-label text classification. Two reasons can explain this finding. On one hand, the fusion of word-level and document-level vectors to represent the original features of the input document increases the length of the document, which then inevitably contains more information. On the other hand, with the increase in the size of the fusion vector, more hidden multi-label features are provided; thus, with the advantage of TextCNN in terms of feature extraction, the effect of multi-label text classification is further improved. Therefore, the following comparative experiments only compare tALBERT-CNN-S3 with other advanced models.

Tables 7, 8, 9 show the comparison results of our proposed model and other basic models and excellent models. On the whole, aside from the better individual evaluation metrics of the LSAN model on the RCV1 dataset, our model effect is relatively excellent, on the whole, while the CNN-based model has the worst effect; this is related to the static Word2vec word-level vectors used by TextCNN and the use of XML-CNN as the original semantic vector representation of the document. Because Word2vec is based on static word vectors, once the model is trained according to the given corpus, the meaning of each word will not change; that is, t if the word is not placed in context, the problem of polysemy cannot be solved.

The LSAN model performs better than our model in terms of some metrics on the RCV1 dataset; this is mainly because the LSAN transforms the label set into a semantic vector and then obtains multi-label text features through a similarity comparison with document semantic vectors. However, this method relies heavily on the given label sets, and only when the number of labels is large, it can fully show its advantages. Our model achieves good results on three different datasets and has stronger applicability than the competing approaches. Especially on the AAPD and IMDB, our model is obviously better than other models, and on RCV1, our model is also better when the evaluation metrics are SA and HL.

## Conclusions

To solve the multi-label text classification problem, this paper proposes a multi-label text classification method that combines document representations of topic information and deep semantic information with a multi-label learning model based on TextCNN. We perform many experiments on three benchmark datasets and explore the influence of the fusion of different levels of topic information and deep semantic information on multi-label text classification. In short, the strategy of fusing topic information and deep semantic information at the word level and document level can achieve the best performance. In addition, to further verify the effectiveness of our proposed method, we also compare it with the excellent methods based on RNNs, CNNs, and the combination of an attention mechanism and a topic model. Aside from the LSAN model being superior to our method in terms of some evaluation metrics on a specific dataset, our method based on the tALBERT-CNN multi-label text classification approach has achieved the best performance, and our method has better applicability than competing approaches. Although our method has achieved good performance on three standard datasets and alleviated the common tail label problem in multi-label classification to a certain extent, we did not propose a thorough solution to the tail label problem. This is also the direction of our continued efforts.

Moreover, we also analyze the characteristics of the LSAN model; that is, the label set is represented by a semantic vector, an attention mechanism is adopted so that the model can learn the similarities between document semantics and label semantics, and then, multi-label classification is carried out. Therefore, we will pay more attention to how to improve the performance of our multi-label model text classification using the similarities between label semantics and document semantics and an attention mechanism based on the transformer architecture.

In addition, solving the problem of few sample classification through meta-learning is also an effective method to solve the tail label problem, which has been favored by scholars in recent years. In fact, our team is currently studying how to use meta-learning to solve the problem of few-shot text classification. This method is mainly aimed at the problems of more text label categories and fewer dataset instances. At present, our research has also made some progress.

## Footnotes

1 Denote the text depth feature representation based on distributed representation and word embedding features, which is realized by the combination of the LDA topic model and ALBERT.

2 Based on the text depth feature representation, TextCNN method is used to extract the text depth feature again.
